# The attenuated incretin effect is associated with glucose intolerance in patients with hepatitis B-related acute-on-chronic liver failure

**DOI:** 10.3389/fphys.2026.1794931

**Published:** 2026-03-27

**Authors:** Meichuan Li, Han Hu, Yujuan Liu, Xiaohuan Wu, Yin Zhang, Ying Li, Fangwan Yang, Yan Li, Shide Lin

**Affiliations:** 1 Department of Infectious Diseases, Affiliated Hospital of Zunyi Medical University, Zunyi, Guizhou, China; 2 Department of Infectious Diseases, Second Affiliated Hospital of Zunyi Medical University, Zunyi, Guizhou, China; 3 Department of Epidemiology and Health Statistics, School of Public Health, Zunyi Medical University, Zunyi, Guizhou, China

**Keywords:** acute-on-chronic liver failure, diabetes mellitus, glucagon-like peptide-1, glucose-dependent insulinotropic polypeptide, incretin effect, insulin

## Abstract

**Objective:**

The pathogenesis of glucose homeostasis disturbance in acute-on-chronic liver failure (ACLF) remains poorly defined. This study aimed to investigate the association between incretin effect (IE) and glucose intolerance in patients with hepatitis B virus-related ACLF (HBV-ACLF).

**Methods:**

In this prospective study, 25 patients with HBV-ACLF were stratified by glucose tolerance: normal glucose tolerance (NGT, n=7), pre-diabetes (pre-DM, n=9), and diabetes mellitus (DM, n=9). All participants underwent paired oral (OGTT) and intravenous glucose tolerance test (IVGTT) within 72 hours of admission. Dynamic profiles of glucagon-like peptide-1 (GLP-1), glucose-dependent insulinotropic polypeptide (GIP), and insulin were analyzed. The IE, calculated from insulin area under the curve (AUC) differences, was compared across groups.

**Results:**

Patients with pre-DM and DM exhibited significantly reduced insulin sensitivity and early-phase insulin secretion, alongside elevated insulin resistance, compared to the NGT group. While GLP-1 and GIP responses post-OGTT remained significantly higher than post-IVGTT across all groups, the GIP secretory response was markedly blunted in DM patients compared to those with NGT. Critically, the incremental insulin response during OGTT versus IVGTT was abolished in DM patients but preserved in pre-DM and NGT. Consequently, IE was progressively and significantly reduced from NGT (58.5 ± 9.2%) to pre-DM (44.5 ± 7.5%) to DM (25.8 ± 8.7%) (*P* < 0.01). These differences remained statistically significant after adjustment for age, sex, body mass index, Model for End-Stage Liver Disease-Sodium score and C-reactive protein.

**Conclusion:**

The attenuated IE is associated with the severity of glucose intolerance, and the development of DM is associated with a blunted GIP secretory response in patients with HBV-ACLF.

## 1 Introduction

Acute-on-chronic liver failure (ACLF) is a clinical syndrome characterized by acute decompensation of chronic liver disease, multi-organ dysfunction, and a high risk of short-term mortality (Bajaj et al., 2022). In China, the predominant etiology is chronic hepatitis B virus (HBV) infection, defining HBV-ACLF. A core pathophysiological driver of ACLF progression is severe hepatic and systemic inflammation (Sarin et al., 2019).

Severe liver dysfunction results in disturbance of glucose homeostasis, manifested as diabetes mellitus (DM) or pre-DM (Coman et al., 2021). Our previous work established that DM significantly elevates mortality in ACLF (Hu et al., 2021). Our previous studies also identified that both relative insulin secretory deficiency and insulin resistance (IR) contribute to glucose dysregulation in cirrhosis and ACLF (Liu et al., 2024). However, the specific mechanisms underlying insufficient insulin secretion in ACLF remain undefined.

The incretin effect (IE) is a critical physiological regulator of postprandial insulin secretion. Upon nutrient ingestion, intestinal L-cells and K-cells secrete glucagon-like peptide-1 (GLP-1) and glucose-dependent insulinotropic polypeptide (GIP), respectively. These hormones potentiate glucose-stimulated insulin secretion from pancreatic β-cells (Müller et al., 2019). Consequently, oral glucose administration elicits a greater insulin response than an isoglycemic intravenous infusion—a phenomenon quantified in clinical studies as the difference in the area under the curve (AUC) of insulin between an oral glucose tolerance test (OGTT) and an intravenous glucose tolerance test (IVGTT) (Aulinger and D'Alessio, 2023).

The IE is markedly diminished in obesity and type 2 diabetes mellitus (T2DM), correlating closely with impaired β-cell function and insulin sensitivity (Nauck and Müller, 2023). In T2DM, the defect often lies not in deficient incretin secretion but primarily in a loss of β-cell responsiveness to GIP, while the response to GLP-1 is relatively preserved (Nauck and Müller, 2023; Accili et al., 2025). Beyond T2DM, an attenuated IE has been implicated in dysglycemia associated with conditions such as psoriasis, critical illness, and non-alcoholic fatty liver disease (Gyldenløve et al., 2015; Nielsen et al., 2015; Junker et al., 2016). Despite this evidence, the role of the incretin system in the disturbed glucose metabolism of ACLF—a state of profound inflammation distinct from stable cirrhosis or T2DM—has not been investigated.

To address this knowledge gap, the present study utilized precisely paired OGTT and IVGTT performed early in the clinical course to evaluate the association between the IE and the spectrum of glucose intolerance in patients with HBV-ACLF.

## 2 Materials and methods

### 2.1 Patient

This prospective study was conducted between October 2022 and December 2024. Consecutively hospitalized patients aged 18–80 years with a diagnosis of HBV-ACLF were recruited from the Departments of Infectious Diseases at the Affiliated Hospital of Zunyi Medical University. The study protocol adhered to the principles of the Declaration of Helsinki and received approval from the institutional Ethics Committee (Approval No.: KLL-2023-244). Written informed consent was obtained from all participants.

### 2.2 Inclusion and exclusion criteria

ACLF was diagnosed according to established criteria: recent onset of jaundice (total bilirubin ≥5 times the upper limit of normal) and coagulopathy (prothrombin activity <40% or international normalized ratio ≥1.5), complicated within 4 weeks by ascites and/or hepatic encephalopathy in patients with underlying chronic liver disease (Sarin et al., 2019). HBV-ACLF was defined as ACLF in patients with a positive hepatitis B surface antigen for more than six months.

From an initial screening of 74 eligible patients, the following exclusions were applied: 8 patients with comorbid hepatocellular carcinoma, severe anemia, or a pre-existing diagnosis of DM; 21 patients with severe extrahepatic complications at admission (e.g., acute variceal bleeding, overt hepatic encephalopathy) or who declined to participate; and 5 patients co-existed with alcoholic liver disease or with previous or present steroid consumption. Consequently, 40 patients successfully completed the OGTT.

**Figure 1:**
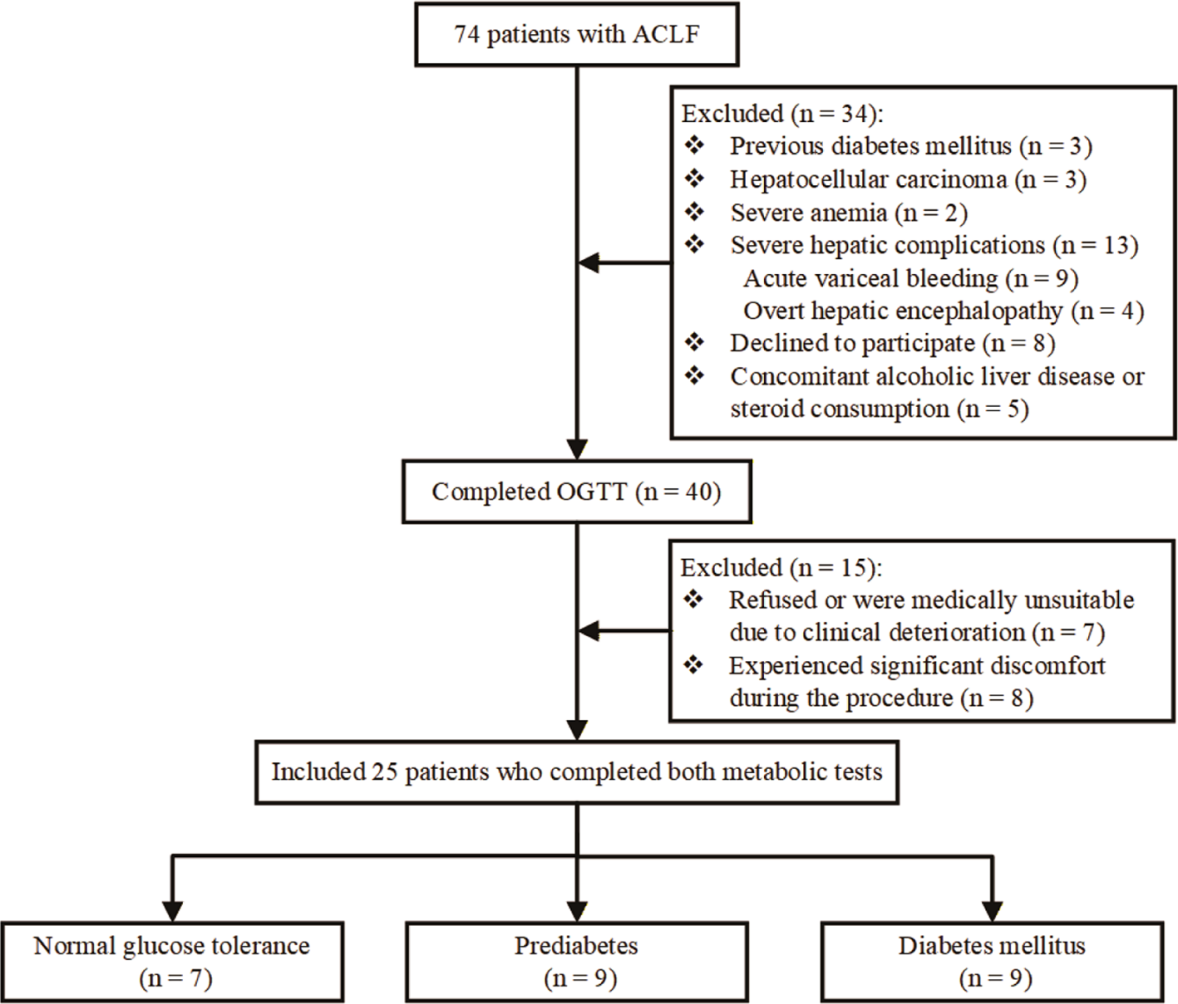
The flowchart of the patient enrollment.

Among these, paired IVGTT were subsequently performed. Further exclusions included 7 patients who refused or were medically unsuitable due to clinical deterioration, and 8 patients who experienced significant discomfort during the procedure. Thus, the final cohort for comparative IE analysis comprised 25 patients who completed both metabolic tests: 7 with normal glucose tolerance (NGT), 9 with pre-diabetes (pre-DM), and 9 with newly diagnosed DM. A flowchart summarizing the patient enrollment process is presented in **Figure 1**.

### 2.3 Metabolic testing procedures

All tests were performed after an overnight fast of 6–8 hours.

#### 2.3.1 OGTT

Following collection of a baseline blood sample (0 h), participants ingested 82.5 g of hydrated glucose (equivalent to 75 g anhydrous glucose) dissolved in 200 mL of water within 3 minutes. Subsequent blood samples were obtained at 0.5, 1, 2, and 3 hours.

#### 2.3.2 IVGTT

The IVGTT was performed within 48 hours after the OGTT. At 8:00 AM, an intravenous catheter was placed in one antecubital vein for glucose infusion, and another cannula was inserted into the contralateral antecubital vein for blood sampling. A bolus of 50% glucose solution (0.5 g/kg body weight) was administered over 3 minutes to rapidly elevate blood glucose levels. Blood samples were collected at identical time points (0, 0.5, 1, 2, and 3 h) for analysis. Adverse events were monitored and managed promptly throughout the procedure.

Blood samples were collected into ethylenediaminetetraacetic acid (EDTA)-coated tubes containing aprotinin (Trasylol; Bayer HealthCare, Leverkusen, Germany) at a concentration of 5000 IU/mL of blood as a dipeptidyl peptidase-4 (DPP-4) inhibitor. The samples were immediately centrifuged, and the separated plasma was stored at −80 °C until analysis.

Plasma concentrations of total GLP-1 and total GIP were measured using enzyme-linked immunosorbent assay (ELISA). The kits were procured from Crystal Chem, Inc. (Elk Grove Village, IL, USA). Total GLP-1 (including forms 1–36, 7–36, and 9–36 amide) was measured using the GLP-1 kit (Cat. #81506). Total GIP was measured using the GIP kit (Cat. #81515).

### 2.4 Diagnostic and analytical criteria

Glucose tolerance status was defined using American Diabetes Association criteria (Committee, 2023): DM as fasting plasma glucose (FPG) ≥7.0 mmol/L or 2-hour plasma glucose (2h-PG) ≥11.1 mmol/L; pre-DM as impaired fasting glucose (FPG 5.6–6.9 mmol/L) and/or impaired glucose tolerance (2h-PG 7.8–11.0 mmol/L). Glycated hemoglobin (HbA1c) was not used for diagnosis due to its known unreliability in advanced liver disease (Ogawa et al., 2022).

Total AUC for glucose, insulin, C-peptide, GLP-1, and GIP was calculated over 3 hours using the trapezoidal method.

The IE was calculated as the ratio of total insulin responses during the OGTT to those during the IVGTT and expressed as a percentage: IE% = 100 × (AUC of insulin OGTT − AUC of insulin IVGTT)/AUC of insulin OGTT (Marini et al., 2012).

The Matsuda index was calculated as: 10,000/√[fasting glucose (mmol/L) × fasting insulin (pmol/L) × mean OGTT glucose × mean OGTT insulin] (Matsuda and DeFronzo, 1999). Homeostasis model assessment 2 indices for insulin resistance (HOMA2-IR) and β-cell function (HOMA2-β) were derived as previously described (Liu et al., 2024).

As previous studies have demonstrated delayed and blunted glucose-stimulated insulin secretion in patients with liver cirrhosis (Greco et al., 2002), early-phase insulin secretion was assessed using insulinogenic index (IGI): IGI = (insulin at 60min − fasting insulin)/(glucose at 60min − fasting glucose).

The Model for End-Stage Liver Disease (MELD) Na score was calculated to evaluate disease severity (Biggins et al., 2006).

### 2.5 Statistical analysis

Data were analyzed using R statistical software (version 4.4.2) within the RStudio environment (version 2024.09.0 + 375). Continuous variables are presented as mean ± standard deviation if normally distributed with homogeneous variance, or as median (interquartile range) otherwise. Normality and homogeneity of variance were assessed using Shapiro-Wilk and Levene’s tests, respectively.

Group comparisons were performed using one-way analysis of variance (ANOVA) with Tukey’s *post-hoc* test for parametric data, Welch’s ANOVA with Games-Howell *post-hoc* test for data with non-homogeneous variance, or the Kruskal-Wallis H test with Dunn’s *post-hoc* test (Bonferroni-adjusted) for non-parametric data. All tests were two-tailed, and a *P*-value < 0.05 was considered statistically significant.

Correlations between IE and disease severity, organ injury, and inflammatory markers were assessed using Spearman’s rank correlation. Results are presented as Spearman’s correlation coefficients (ρ) with corresponding two-sided *P* values.

Linear regression models were employed to examine the association between glucose tolerance group and IE, with IE specified as the dependent variable. Crude and adjusted models were constructed. Pairwise comparisons between glucose tolerance groups were performed using estimated marginal means (EMMs) with a Tukey adjustment for multiple comparisons. All inferences in the regression analyses were based on HC3 heteroscedasticity-consistent standard errors. A two-sided *P* < 0.05 was considered statistically significant.

## 3 Results

### 3.1 Baseline clinical and biochemical characteristics

The baseline characteristics of the study cohort stratified by glucose tolerance status (NGT, pre-DM, and DM) are summarized in **Table 1**. Patients with DM had significantly higher FPG levels than both the NGT and pre-DM groups (*P* < 0.05), and also exhibited significantly higher fasting plasma C-peptide levels compared to the NGT group (*P* < 0.05). No significant differences were observed among the three groups regarding age, sex distribution and body mass index (BMI). Liver function tests, systemic inflammation indices, alpha-fetoprotein, renal function, plasma insulin levels and coagulation profiles were comparable across groups. Disease severity, as assessed by the MELD-Na score, exhibited a non-significant upward trend with worsening glucose metabolism (*P* = 0.201). These findings indicate that the study groups were well-matched in terms of underlying liver disease severity and systemic inflammatory burden.

**Table 1:** Clinical characteristics of patients with ACLF and glucose intolerance.

Variable	NGT (*n_1_* = 7)	pre-DM (*n_2_* = 9)	DM (*n_3_* = 9)	*P* value
Men (n%)	5 (71.4)	7 (77.8)	8 (88.9)	0.829
Age (years)	41.6 ± 15.6	46.8 ± 14.5	55.7 ± 12.1	0.145
BMI (kg/m^2^)	20.9 ± 4.7	22.6 ± 3.3	24.7 ± 3.4	0.149
Na^+^ (mmol/L)	136.9 (135.5, 138.1)	136.8 (136.0, 139.3)	138.0 (131.4, 139.0)	0.924
ALT (U/L)	406.0 (127.0, 695.0)	140.0 (131.0, 155.0)	157.0 (121.0, 198.0)	0.601
AST (U/L)	210.0 (190.5, 403.0)	149.0 (141.0, 158.0)	133.0 (108.0, 183.0)	0.153
ALB (g/L)	32.8 ± 6.1	29.2 ± 6.3	28.0 ± 5.3	0.278
GGT (U/L)	118.0 (83.5, 200.5)	54.0 (32.0, 76.0)	97.0 (47.0, 143.0)	0.207
ALP (U/L)	143.0 (135.0, 186.5)	126.0 (72.0, 182.0)	158.0 (139.0, 224.0)	0.213
TBIL (μmol/L)	110.6 (99.2, 167.6)	102.5 (89.6, 301.1)	107.3 (98.6, 253.0)	0.959
PTA (n%)	33.8 ± 8.9	25.9 ± 9.9	32.8 ± 8.8	0.188
INR	1.86 ± 0.13	2.08 ± 0.28	2.19 ± 0.39	0.107
AFP (ng/ml)	28.1 (17.7, 49.3)	6.50(3.8, 64.1)	6.9 (3.5, 39.7)	0.612
MELD-Na	19.9 (19.3, 22.9)	21.9 (20.8, 27.7)	26.7 (20.4, 29.4)	0.201
Cr (μmol/L)	73.0 (65.5, 76.5)	79.0 (77.0, 92.0)	90.0 (76.0, 127.0)	0.113
CRP (mg/L)	6.8 (4.6, 13.2)	18.2 (4.4, 25.5)	13.1 (3.4, 32.9)	0.612
PCT (ng/ml)	0.19 (0.11, 0.43)	0.40 (0.08, 0.67)	0.27 (0.16, 0.74)	0.835
WBC (×10^9^/L)	4.91 ± 1.59	6.30 ± 3.03	6.26 ± 2.05	0.440
HGB (g/L)	124.9 ± 26.8	105.1 ± 25.9	120.1 ± 29.9	0.335
PLT (×10^9^/L)	151.1 ± 59.0	101.2 ± 51.8	97.3 ± 50.4	0.115
FPG (mmol/L)	4.25 (4.07, 5.05)	4.28 (3.79, 4.48)	5.51 (5.06, 7.28)^#※^	0.039
FPI (μIU/ml)	7.67 ± 3.87	7.85 ± 5.68	12.51 ± 7.30	0.182
FPC (pmol/L)	835.3 (744.8, 852.3)	987.00 (661.0, 1212.0)	1281.0 (1010.0, 1432.0)^#^	0.032

^#^DM vs. NGT; ^※^DM vs. pre-DM. Data are presented as n (%), mean ± standard deviation, or median (interquartile range).

ACLF, acute-on-chronic liver failure; AFP, alpha-fetoprotein; ALB, albumin; ALP, alkaline phosphatase; ALT, alanine aminotransferase; AST, aspartate aminotransferase; BMI, body mass index; Cr, creatinine; CRP, C-reactive protein; DM, diabetes mellitus; FPC, fasting plasma C-peptide; FPG, fasting plasma glucose; FPI, fasting plasma insulin; GGT, gamma-glutamyl transferase; HGB, hemoglobin; INR, international normalized ratio; MELD-Na, Model for End-Stage Liver Disease-Sodium score; Na^+^, serum sodium; NGT, normal glucose tolerance; PCT, procalcitonin; PLT, platelet count; pre-DM, prediabetes; PTA, prothrombin time activity; TBIL, total bilirubin; WBC, white blood cell count.

### 3.2 Insulin sensitivity, secretion, and resistance in patients with ACLF

**Figure 2:**
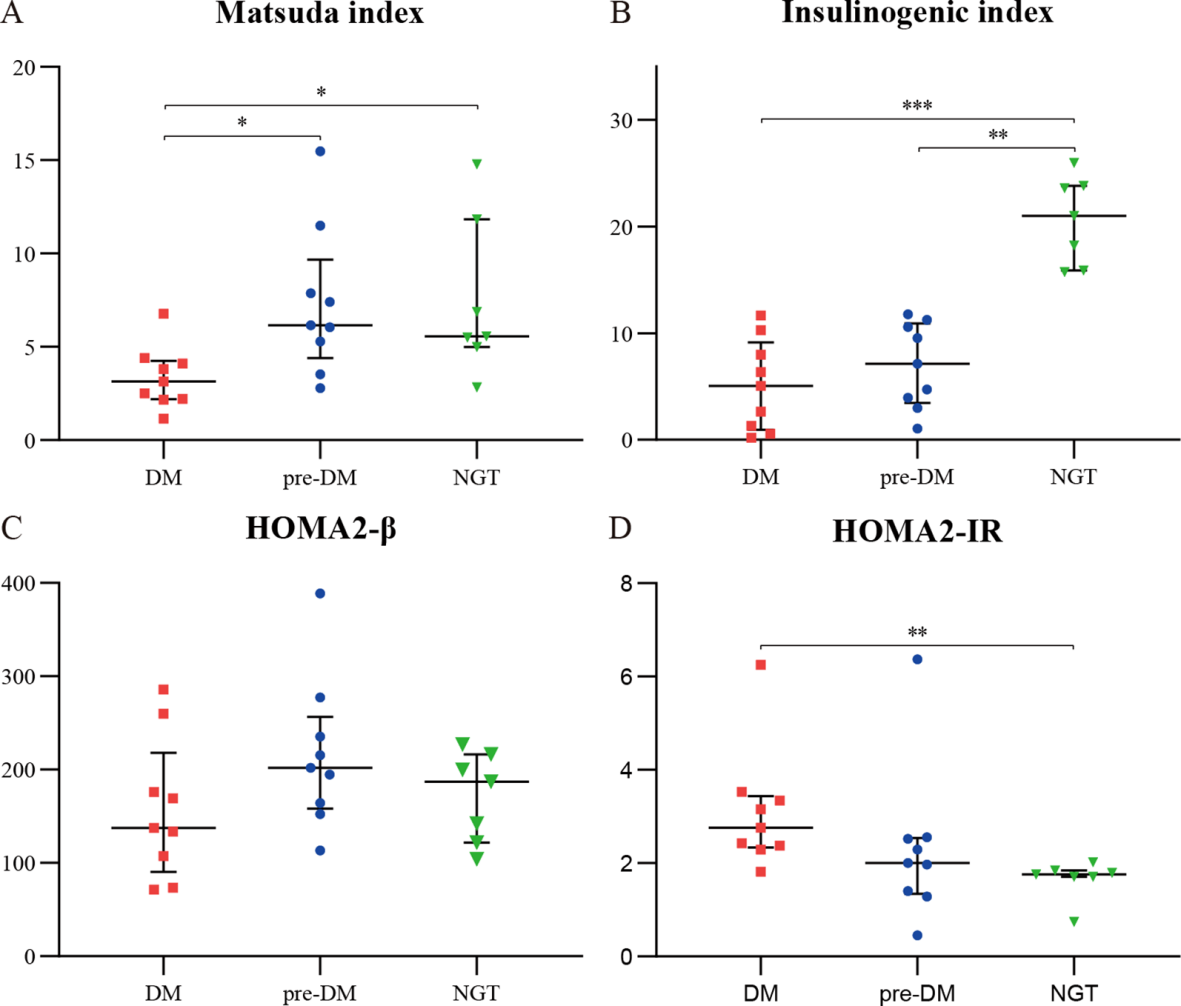
Insulin sensitivity, secretion, and resistance indices in patients with ACLF. **(A)** Matsuda index; **(B)** insulinogenic index (IGI); **(C)** HOMA2-β; **(D)** HOMA2-IR. Data are presented as median (interquartile range). DM, diabetes mellitus; NGT, normal glucose tolerance; pre-DM, prediabetes. * P <0.05; ** P <0.01; *** P<0.001.

As illustrated in **Figure 2**, insulin sensitivity, assessed by the Matsuda index, was significantly lower in the DM group compared to both the pre-DM and NGT groups (all *P* < 0.05). Early-phase insulin secretion, quantified by the IGI, was markedly impaired in both the pre-DM and DM groups relative to the NGT group (*P* < 0.01 and *P* < 0.001, respectively), with no significant difference observed between the pre-DM and DM groups. While β-cell function (HOMA2-β) showed a declining trend from NGT to DM, this did not reach statistical significance. Insulin resistance (HOMA2-IR) was significantly elevated in the DM group compared to the NGT group (*P* < 0.05).

### 3.3 Glucose, insulin, and C-peptide dynamics

**Figure 3:**
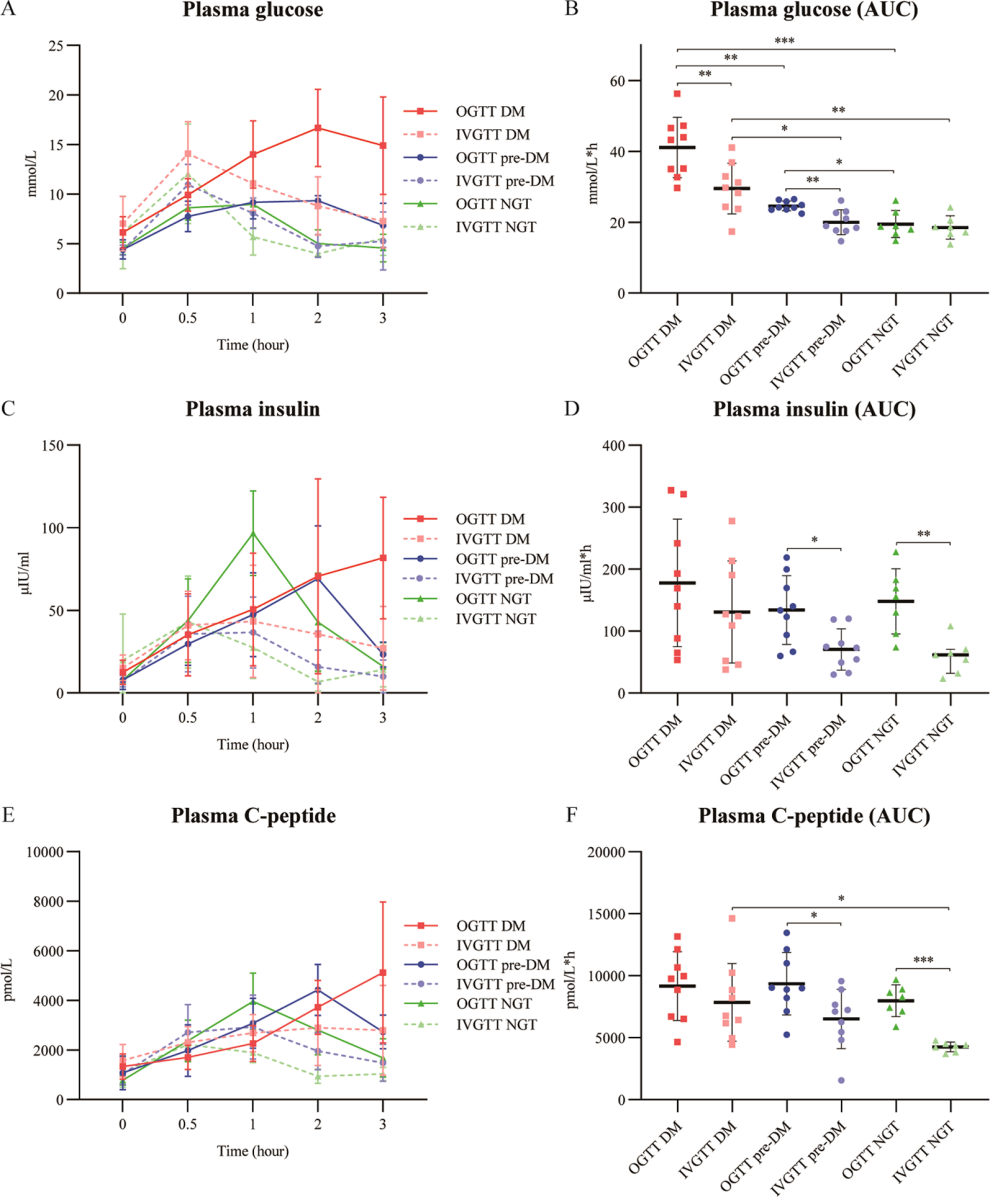
Dynamic profiles of glucose, insulin, and C-peptide in patients with ACLF during oral and intravenous glucose tolerance tests. Time-course curves and corresponding AUC values for plasma glucose **(A, B)**, insulin **(C, D)**, and C-peptide **(E, F)** in patients stratified by glucose tolerance status (NGT, pre-DM, and DM). Data are presented as mean ± standard deviation or median (interquartile range). ACLF, acute-on-chronic liver failure; AUC, area under the curve; DM, diabetes mellitus; IVGTT, intravenous glucose tolerance test; NGT, normal glucose tolerance; OGTT, oral glucose tolerance test; pre-DM, prediabetes. * P <0.05; ** P <0.01; *** P <0.001.

The dynamic responses of glucose, insulin, and C-peptide during OGTT and IVGTT in patients with different glucose intolerance strata are detailed in **Figure 3**.

During OGTT, the time to peak plasma glucose was delayed in the DM group (2 hours) compared to the pre-DM and NGT groups (both at 1 hour). In all groups, plasma glucose peaked at 0.5 hours during IVGTT (**Figure 3A**). The glucose AUC during OGTT was significantly greater than during the matched IVGTT in both the DM and pre-DM groups (both *P* < 0.01), but not in the NGT group (**Figure 3B**). Inter-group comparisons revealed a graded increase in glucose AUC during OGTT (DM > pre-DM > NGT, all *P* < 0.05). During IVGTT, the glucose AUC was higher in the DM group than in both the pre-DM and NGT groups (*P* < 0.05 and *P* < 0.01, respectively).

The insulin secretory pattern differed notably among groups (**Figure 3C**). During OGTT, insulin levels in the DM group exhibited a blunted and progressively rising profile without a clear peak within 3 hours. In contrast, insulin peaked at 2 hours in the pre-DM group and at 1 hour in the NGT group before declining. During IVGTT, all groups showed a rapid insulin peak at 0.5 hours followed by a gradual decline. Critically, the insulin AUC was significantly greater during OGTT than during IVGTT in both the pre-DM and NGT groups (*P* < 0.05), indicating a preserved incretin-mediated insulinotropic effect. This difference was absent in the DM group (**Figure 3D**).

C-peptide dynamics closely mirrored those of insulin during both tests. Notably, during IVGTT, the C-peptide AUC was significantly higher in the DM group than in the NGT group (**Figures 3E, F**).

### 3.4 incretin hormone dynamics

**Figure 4:**
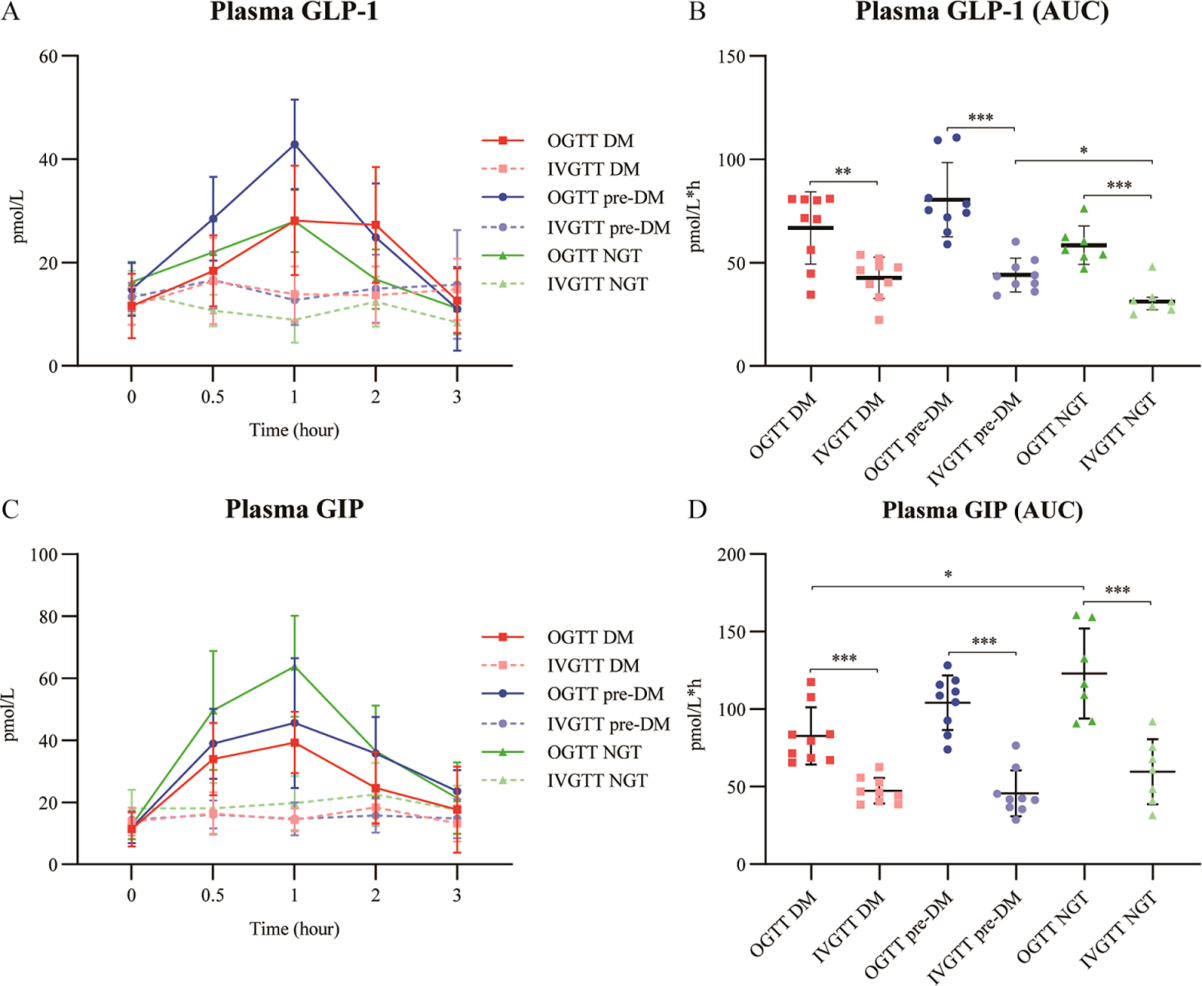
Dynamic profiles of incretin hormones in patients with ACLF during oral and intravenous glucose tolerance tests. Time-course curves and corresponding AUC values for plasma GLP-1 **(A, B)** and GIP **(C, D)** across glucose-tolerance strata (DM, pre-DM, and NGT). Data are presented as mean ± standard deviation or median (interquartile range). ACLF, acute-on-chronic liver failure; AUC, area under the curve; DM, diabetes mellitus; GIP, glucose-dependent insulinotropic polypeptide; GLP-1, glucagon-like peptide-1; IVGTT, intravenous glucose tolerance test; NGT, normal glucose tolerance; OGTT, oral glucose tolerance test; pre-DM, prediabetes. * P <0.05; ** P <0.01; *** P <0.001.

The secretory profiles of GLP-1 and GIP in patients with different glucose intolerance strata are presented in **Figure 4**.

Following OGTT, the GLP-1 response was most pronounced in the pre-DM group (**Figure 4A**). The GLP-1 AUC during OGTT was significantly higher than during IVGTT across all groups (all *P* < 0.05). During IVGTT, the pre-DM group exhibited a higher GLP-1 AUC than the NGT group (**Figure 4B**).

The post-OGTT GIP response was most robust in the NGT group, attenuated in the pre-DM group, and markedly blunted in the DM group (**Figure 4C**). GIP levels remained stable during IVGTT in all groups. Consequently, the GIP AUC during OGTT was significantly higher than during IVGTT (*P* < 0.01), and was significantly lower in the DM group compared to the NGT group during OGTT (*P* < 0.05) (**Figure 4D**).

### 3.5 The incretin effect in relation to glucose tolerance status

**Figure 5:**
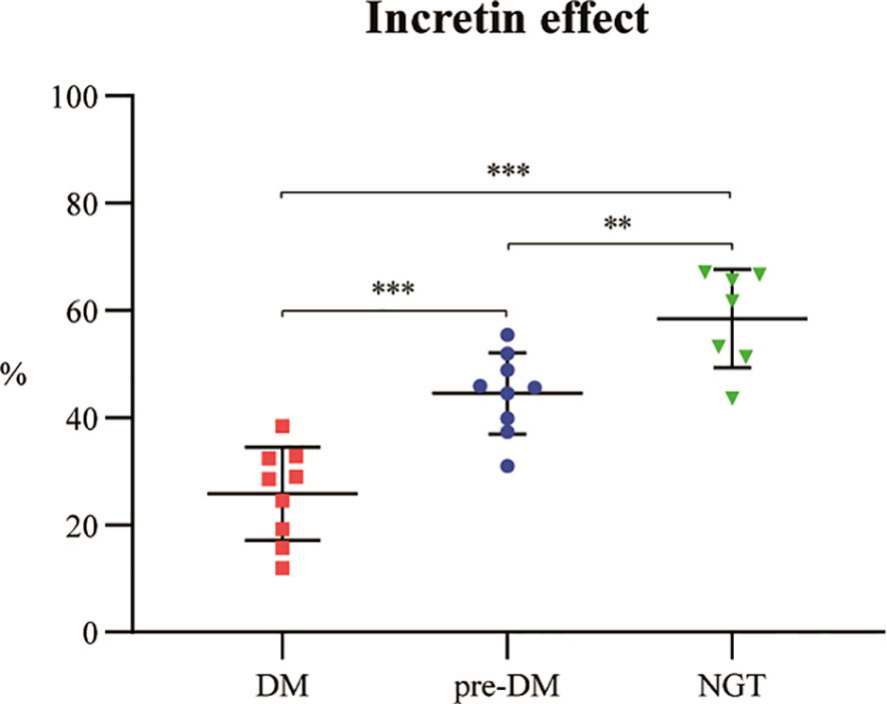
Incretin effect across glucose tolerance strata in patients with ACLF. Data are presented as median (interquartile range). ACLF, acute-on-chronic liver failure; DM, diabetes mellitus; NGT, normal glucose tolerance; pre-DM, prediabetes. ** P <0.01; *** P <0.001.

The calculated IE demonstrated a significant and progressive impairment with worsening glucose intolerance (**Figure 5**). The IE was 58.5 ± 9.2% in the NGT group, 44.5 ± 7.5% in the pre-DM group, and 25.8 ± 8.7% in the DM group. Pairwise comparisons revealed that the IE in the NGT group was significantly higher than in both the pre-DM (*P* = 0.009) and DM groups (*P* < 0.001). Furthermore, the IE in the pre-DM group remained significantly higher than in the DM group (*P* < 0.001).

Spearman’s rank correlation analysis revealed that the IE was not significantly correlated with the MELD-Na score, total bilirubin, C-reactive protein levels (CRP), or white blood cell count (all *P* > 0.05, data not shown). Pairwise differences in the IE, with 95% confidence intervals (CIs), derived from linear regression models, are presented in **Table 2**. *Post hoc* comparisons of the IE across glucose tolerance groups demonstrated that the differences among groups remained statistically significant after adjustment for age, sex, BMI, MELD-Na score and CRP (all *P* < 0.05).

**Table 2:** *Post hoc* comparisons of the incretin effect across glucose tolerance groups.

Comparison	Unadjusted		Adjusted*	
	Difference in IE (95% CI)	*P* value	Difference in IE (95% CI)	*P* value
NGT vs. pre-DM	0.140 (0.045, 0.235)	0.006	0.172 (0.013, 0.331)	0.033
NGT vs. DM	0.326 (0.226, 0.427)	<0.001	0.352 (0.209, 0.496)	<0.001
Pre-DM vs. DM	0.187 (0.102, 0.271)	<0.001	0.181 (0.035, 0.326)	0.014

Data are presented as pairwise differences in the IE with 95% confidence intervals, derived from linear regression models.

*Adjusted estimates include age, sex, BMI, MELD-Na score and CRP as covariates.

BMI, body mass index; CI, confidence interval; CRP, C-reactive protein; DM, diabetes mellitus; IE, incretin effect; MELD-Na, Model for End-Stage Liver Disease-Sodium score; NGT, normal glucose tolerance; pre-DM, prediabetes.

## 4 Discussion

DM occurring secondary to severe liver injury has been defined as hepatogenous diabetes (HD) (Kumar et al., 2022). As HD shares some pathophysiological similarities with T2DM, such as significant IR, hyperinsulinemia, and impaired β-cell function (Garcia-Compean et al., 2016), the World Health Organization has not yet recognized HD as a distinct disease entity. However, compared with T2DM, HD exhibits some distinctive clinical features: HD typically develops after the diagnosis of chronic liver disease and its prevalence correlates directly with the severity of hepatic impairment, suggesting that severe liver injury and the related pathophysiological alterations play a central role in its pathogenesis (Orsi et al., 2016). Therefore, whether HD possesses a unique pathogenic mechanism remains to be elucidated.

The pathophysiology of ACLF is characterized by hepatic impairment and systemic inflammation, both of which can disrupt glucose regulation, leading to HD and stress-induced hyperglycemia. Additionally, some patients may have pre-existing, undiagnosed diabetes. In this study, although we could not definitively exclude the presence of undiagnosed DM, the exclusion of patients with known DM suggests that the observed glucose intolerance was predominantly attributable to the hepatic impairment and systemic inflammation inherent to ACLF.

The principal findings of this study are that glucose intolerance in HBV-ACLF was characterized by a triad of IR, impaired early-phase insulin secretion (low IGI), and a graded attenuation of the IE from NGT through pre-DM to overt DM. Notably, while post-prandial GLP-1 secretion remained intact, the GIP response to oral glucose was significantly blunted in patients with DM.

Our observation of a delayed insulin peak during OGTT, along with a reduced IGI in ACLF-associated DM, further indicates that early-phase insulin insufficiency is a hallmark feature of glucose intolerance in ACLF patients. This finding is consistent with our previous reports (Liu et al., 2024). While early postprandial glucose elevation serves as the primary physiological stimulus for insulin secretion, delayed gastric emptying, a frequent complication in advanced liver diseases (Marathe et al., 2015; Alukal and Thuluvath, 2019), may partly explain the attenuated and prolonged insulin response observed in this population. However, the exaggerated glycemic rise before 3 hours during OGTT we observed in ACLF-DM patients argues against absorptive delay as the primary culprit. Moreover, as shown in **Figure 3**, during the IVGTT, the glucose AUC was significantly higher in the DM group than in the NGT group; however, there was no significant difference in insulin AUC between the two groups. These results suggest that in patients with DM, the increased glucose failed to elicit a proportionately greater insulin response than in NGT. These findings confirm impaired β-cell glucose sensitivity in patients with ACLF-DM.

A key novel finding is the progressive loss of IE in ACLF with worsening glucose tolerance. This progressive reduction in IE remained statistically significant after adjustment for age, sex, BMI, MELD-Na score and CRP. In T2DM, the reduced IE is primarily attributed to diminished β-cell responsiveness to GIP, with relative preservation of GLP-1 action (Ahn et al., 2023). In this study we found that in patients with ACLF-DM, while IE was severely compromised and GLP-1 secretion remained preserved, the GIP response to enteral glucose appeared to be markedly blunted—a finding less commonly observed in T2DM (Chia and Egan, 2020). This suggests that the development of DM is associated with a blunted GIP secretory response in patients with HBV-ACLF.

The parthenogenesis of blunted GIP secretory response in patients with ACLF-DM remains unknown, but it may be partially attributed to the unique pathophysiology of ACLF. The secretion of GIP by K-cells is regulated by multiple factors under both physiological and pathological conditions. Previous studies found that intestinal mucosal injury, gut microbiota dysbiosis, and intestinal hypoxia could impair GIP secretion by K-cells (Lee et al., 2018; Sharma et al., 2024). GIP release is also finely modulated via neural, hormonal, and paracrine pathways; for instance, sympathetic nervous activity, somatostatin, and leptin can inhibit its secretion (Jensen et al., 2012; Yamane et al., 2025). Furthermore, studies had shown that in sepsis, elevated levels of DPP-IV could accelerate the degradation of both GLP-1 and GIP (Iwanaga and Nio-Kobayashi, 2021). Profound enteropathy, severe endocrine and metabolic dysfunction have been found in patients with advanced liver diseases (Alukal and Thuluvath, 2019), driven by portal hypertension, intestinal congestion, mucosal hypoxia, and dysbiosis (Arroyo et al., 2021; Engelmann et al., 2021). Thus, the development of ACLF may specifically disrupt the function of the K-cell.

Nevertheless, a blunted GIP secretory response alone may not fully account for the abolished IE in ACLF-DM. Our study cannot exclude concomitant “incretin resistance”, a diminished insulinotropic potency of both GLP-1 and GIP at the level of the β-cell (Accili et al., 2025). Systemic inflammation, a hallmark of ACLF, is a potent inducer of both β-cell apoptosis and insulin resistance (Bar-Or et al., 2019). Chronic hyperglycemia itself can further desensitize β-cells to incretin stimulation (Rosenberg et al., 2021). Therefore, the observed IE defect likely results from a combination of insufficient GIP signal and impaired β-cell capacity to respond to the remaining incretin tone.

The strengths of this study include the rigorous application of paired, OGTT and IVGTT early in the disease course, minimizing confounding from prolonged hospitalization or nutritional variations in patients with HBV-ACLF. We demonstrated a graded attenuation of the IE from NGT through pre-DM to overt DM, to our knowledge, this is the first report to quantify IE across the spectrum of glucose intolerance in ACLF.

Several limitations of this study should be acknowledged. First, we did not perform isoglycemic clamps during the IVGTT, resulting in differing glycemic stimuli between the oral and intravenous glucose challenges. However, as illustrated in **Figure 3**, blood glucose levels during the OGTT were higher than those during the IVGTT. Consequently, our calculation may have overestimated the IE, since the OGTT-induced insulin secretion was likely potentiated by a stronger glycemic stimulus in addition to gut-derived signals.

Second, due to the clinical characteristics of instability inherent to ACLF, most patients with serious complications were excluded, resulting in a relatively small sample size and potential selection bias of this study. Nonetheless, it was sufficient to demonstrate significant differences in key parameters. Due to the modest sample size, the potential for Type II error should be considered when interpreting the non-significant findings. Thus, our findings may be generalizable only to relatively stable patients with HBV-ACLF who are able to undergo both OGTT and IVGTT. Given that glucose intolerance is associated with a higher prevalence of cirrhosis-related complications (Nishida et al., 2006), it is plausible that patients with more severe complications might exhibit even greater disturbances in glucose homeostasis than those observed in our cohort. Therefore, our findings may represent an underestimation of the true metabolic derangements in the broader ACLF population.

Third, we did not assess gastrointestinal motility, intestinal integrity, nutritional status, or plasma DPP-4 activity, which limits our ability to elucidate the underlying mechanisms contributing to the attenuated IE. Moreover, in the context of ACLF, factors such as severe hepatic impairment, decreased gastrointestinal motility, and systemic inflammation may compromise the accuracy of using OGTT and IVGTT to evaluate IE. Future investigations incorporating these parameters are warranted.

Finally, given the limited number of patients included, we were unable to analyze the association between the IE and clinical outcomes in patients with ACLF. Multi-center studies with larger cohorts are warranted to validate and extend our findings in the future.

## 5 Conclusion

In patients with HBV-ACLF, worsening glucose intolerance is strongly associated with a progressively impaired IE. This defect is characterized by a blunted GIP secretory response, potentially stemming from ACLF-specific intestinal and systemic pathologies. These findings shed new light on the pathophysiology of diabetes in ACLF and suggest that therapeutic strategies targeting the incretin axis, particularly GIP signaling, warrant investigation in this high-risk population.

## Data Availability

The original contributions presented in the study are included in the article/supplementary material. Further inquiries can be directed to the corresponding author.
